# Relationship between hemoglobin and insulin-like growth factor-1 in children and adolescents with idiopathic short stature

**DOI:** 10.1186/s12902-020-00600-w

**Published:** 2020-08-03

**Authors:** Qianqian Zhao, Mei Zhang, Baolan Ji, Yuntian Chu, Hui Pan, Wenhua Yan, Bo Ban

**Affiliations:** 1 Department of Endocrinology, Affiliated Hospital of Jining Medical University, Jining Medical University, 89 Guhuai Road, Jining, Shandong 272029 P.R. China; 2 Chinese Research Center for Behavior Medicine in Growth and Development, 89 Guhuai Road, Jining, Shandong 272029 P.R. China; 3 School of Health Management and Medicine, Tongji Medical College, Huazhong University of Science and Technology, Wuhan, Hubei 430030 P.R. China; 4 Key Laboratory of Endocrinology of National Health and Family Planning Commission, Department of Endocrinology, Peking Union Medical College Hospital, Chinese Academy of Medical Science and Peking Union Medical College, Beijing, 100730 P.R. China

**Keywords:** Insulin-like growth factor-1, Hemoglobin, Idiopathic short stature

## Abstract

**Background:**

The growth hormone/insulin-like growth factor-1 (GH/IGF-1) axis is critical for the regulation of children’s growth and development. Serum IGF-1 concentrations are usually low in individuals with idiopathic short stature (ISS) despite normal endogenous GH levels, and the associated underlying factors are unknown. This study aimed to explore the relationship between IGF-1 and hemoglobin (Hb) in children with ISS.

**Methods:**

A cross-sectional analysis was performed including 178 children and adolescents with ISS who were enrolled from March 2013 to February 2019. The related clinical and biochemical parameters were evaluated for each patient. Univariate analysis, smooth curve fitting and multivariate piecewise linear regression were performed.

**Result:**

The mean levels of IGF-1 standard deviation scores (SDS) and Hb were − 0.99 (− 1.60 - -0.09) and 131.81 ± 9.36 g/L, respectively. Univariate analysis displayed a significant positive association between Hb and IGF-1 SDS (*P* < 0.001). After adjusting for potential confounding factors, the positive relationship between Hb and IGF-1 SDS remained (*P* = 0.001). Furthermore, there was an inflection point for Hb in the curve. In a multivariate piecewise linear regression model, IGF-1 SDS was significantly positively associated with Hb when Hb concentrations were lower than 145 g/L (B 0.05; 95% CI 0.02, 0.07; *P* < 0.001). However, IGF-1 SDS decreased with increasing Hb levels when Hb concentrations were greater than 145 g/L (B -0.15; 95% CI -0.23, − 0.06; *P* = 0.001).

**Conclusion:**

This study demonstrated that Hb is associated with IGF-1 in Chinese children and adolescents with ISS. The levels of IGF-1 increased with the elevation of Hb, but when the concentration of Hb exceeded a certain range, with the increase of Hb, IGF-1 decreased instead.

## Background

Idiopathic short stature (ISS) is defined by a height more than two standard deviation scores (SDS) below the median height for the relevant age and sex of the subject for whom, with the currently available diagnostic tools, no etiology has been established [1]. Although there are no clear causes, several studies have attempted to identify potential mechanisms for impaired linear growth in children with ISS [2, 3]. The growth hormone/insulin-like growth factor-1 (GH/IGF-1) axis is central to the regulation of child growth and development during critical periods. Many children with ISS have low IGF-1 levels despite normal endogenous GH levels [4]. A previous study has shown that a substantial proportion of children with ISS had an impaired GH/IGF-1 axis [5].

Therefore, investigating factors associated with IGF-1 can contribute to understanding the underlying mechanisms of ISS. We previously reported that uric acid (UA) and low-density lipoprotein cholesterol (LDL-C) are associated with IGF-1 [6, 7]. In the present study, we explored the association of hemoglobin (Hb) levels with IGF-1. Many studies have demonstrated that Hb is significantly associated with the linear growth of children [8]. There are certain long-term complications for childhood with low concentrations of Hb, particularly leading to curbed growth [9]. However, the mechanism by which Hb may be related to linear growth is largely unknown. One possible mechanism could be that Hb improves tissue oxygenation, which subsequently enhances optimal cell proliferation and physical growth [10, 11]. Soliman et al. pointed out the possible relationship between low concentrations of Hb and impaired IGF-1 [12]. On the other hand, excessive Hb levels also have adverse effects on children’s growth and development. High Hb concentrations are a risk factor for hypertension and adverse pregnancy outcomes. Potential mechanisms include oxidative stress, increased blood viscosity, and impaired systemic response to inflammation and infection [13–15], which might negatively impact the linear growth of children [16]. However, no study has focused on investigating the relationship between high Hb concentrations and growth indicators.

Recent studies have demonstrated the relationships between IGF-1 levels and Hb in different populations, including nondiabetic adults [17], aged adults [18] and children with premature adrenarche (PA) [19]. Given that both IGF-1 and Hb are associated with childhood growth and development, an association analysis between IGF-1 and Hb in children with short stature may be helpful to further elucidate the pathogenesis of short stature. Unfortunately, at present, a literature review of the relationship between Hb and IGF-1 in ISS has not been performed. The present study aimed to investigate the relationship between Hb and IGF-1 in children and adolescents with ISS.

## Methods

### Subjects

**Fig. 1:**
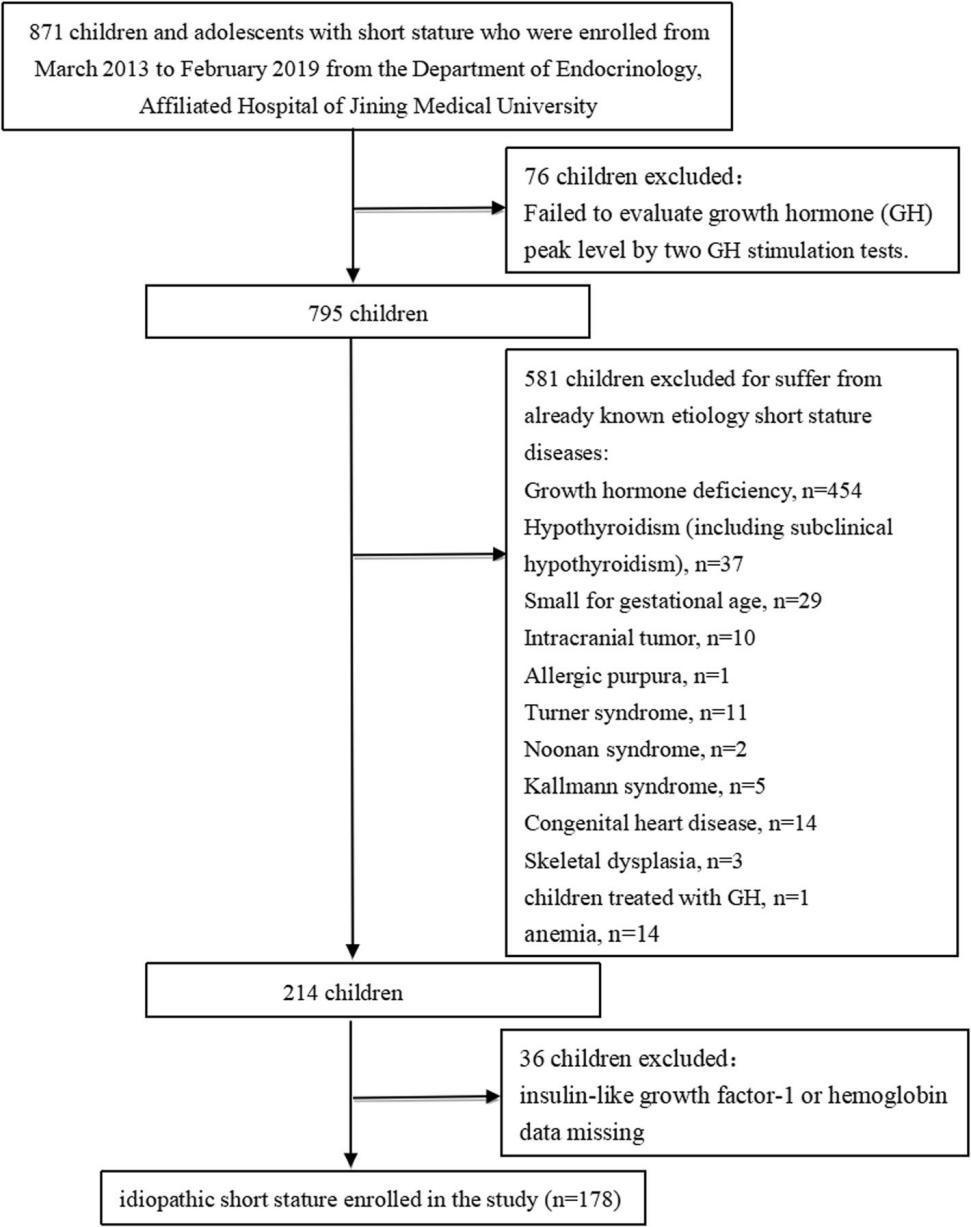
Flow chart of the study population

The subjects were enrolled from March 2013 to February 2019 from the Department of Endocrinology, Affiliated Hospital of Jining Medical University. The subjects were part of the GDDSD study (Growth and Development Diseases in Shandong Province: a cohort follow-up study, http://www.chictr.org.cn, ChiCTR1900026510). Data for the analysis were extracted from the Affiliated Hospital of Jining Medical University information system. A total of 178 children and adolescents with ISS (138 males and 40 females) with an average age of 10.4 ± 3.8 years were enrolled. The subjects with height SDS lower than or equal to − 2 SD after adjusting for age and sex, an appropriate birth weight for gestational age, and GH sufficiency confirmed by a GH concentration > 10 ng/mL in at least two GH provocative tests were included in the study. The exclusion criteria included children with GH deficiency, skeletal dysplasia, thyroid dysfunction, chronic anemia or other known causes of short stature, including Prader-Willi syndrome, Turner syndrome or having been small for gestational age. In addition, children treated with medication interfering with GH secretion or its action were also excluded. The flow chart of the study selection process is shown in Fig. 1.

### Anthropometry measurements

All anthropometric measurements were obtained according to standard procedures. Height was measured to the nearest 0.1 cm without shoes using a stadiometer (Nantong Best Industrial Co., Ltd., Jiangsu, China). Weight was measured by an electronic scale to the nearest 0.1 kg (Wuxi Weigher Factory Co., Ltd., Jiangsu, China) while the individual was wearing light clothes without shoes. Height SDS were expressed using the growth curve of Chinese children as a reference [20]. Body mass index (BMI) was calculated as the ratio of the weight divided by the square of the height in meters. Puberty stage was evaluated by physical examination based on the Tanner stages [21]. Individuals meeting the following criteria were considered prepubescent: boys with no pubic hair and a testicular volume less than 4 mL and girls with no pubic hair and no breast development.

### Laboratory measurements

Fasting blood samples were obtained from all subjects to measure laboratory parameters. Two stimulating tests were performed for GH (500 mg of levodopa for those weighing more than 30 kg and 250 mg of levodopa for those weighing less than 30 kg, orally, and 0.1 U/kg insulin, subcutaneously). A chemiluminescence method was used to assess serum GH concentrations (Access 2, Beckman Coulter, USA), and the sensitivity was 0.010 μg/L. Serum IGF-1 was measured by a chemiluminescence assay (DPC IMMULITE 1000 analyzer, SIEMENS, Germany) with intra- and interassay coefficients of variation (CVs) of 3.0 and 6.2%, respectively. Hb was measured by an automatic blood analyzer (XN-20 (Al), SYSMEX, Japan). Fasting plasma glucose (FPG), kidney function (including creatinine (Cr), blood urea nitrogen (BUN) and UA), Alanine aminotransferase (ALT) and lipid profiles (including triglycerides (TG), total cholesterol (TC), high-density lipoprotein cholesterol (HDL-C), and LDL-C) were determined by an automatic biochemical analyzer (Cobas c702, Roche, Shanghai, China). Measures of estradiol and testosterone were tested by a luminescence immunoassay system (Cobas e 602, Roche, Shanghai, China). IGF-1 SDS were calculated based on IGF-1 levels matched to both age and sex healthy children and adolescents Japanese data [22].

### Statistical analysis

**Fig. 2:**
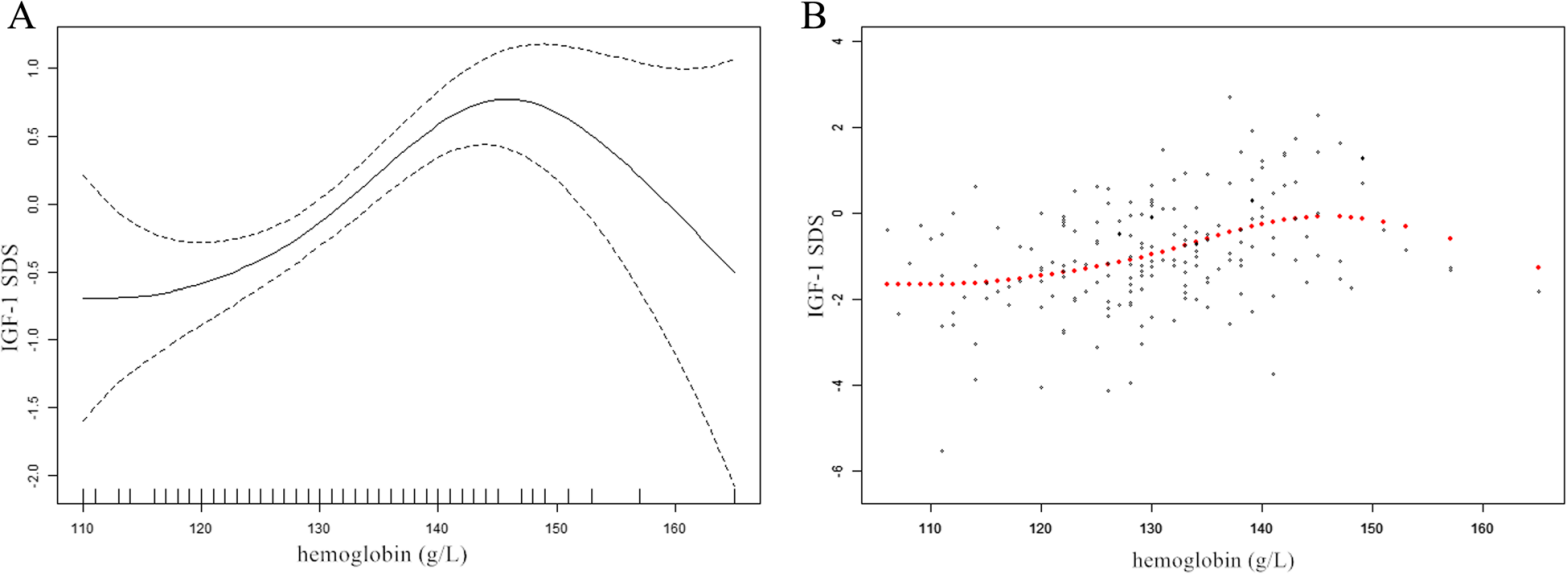
The relationship between Hb and IGF-1 SDS by smooth curve fitting (*n* = 178), *P* < 0.001. The solid line is the curve fitting line, and the dotted line is the 95% confidence interval (**a**). Scatter plot of the distribution of child Hb and IGF-1 SDS (**b**). Adjustment variables: age, height, weight, Cr, UA, FPG, estradiol, testosterone and pubertal stage. Cr: creatinine; UA: uric acid; FPG: fasting plasma glucose

Normally distributed variables are displayed as the mean ± standard deviation, nonnormally distributed variables are displayed as the median (interquartile range), and categorical variables are expressed using a number and percentage (Table 1). Univariate analysis (Table 2) was used to assess whether Hb and other variables were associated with IGF-1 SDS. Multiple linear regression and piecewise linear regression was further used to examine the independent association and threshold effect of IGF-1 SDS and Hb (Table 3). The flow chart of the study selection process is shown in Fig. 1. Smooth curve fitting was used to explore the relationship between IGF-1 SDS and Hb (Fig. 2). A two-tailed *P* < 0.05 was considered statistically significant in all analyses. Statistical analysis was performed with R 3.4.3 (https://www.R-project.org) and EmpowerStats (https://www.empowerstats.com, X&Y Solutions, Inc., Boston, MA).

**Table 1:** Clinical and biochemical characteristics

Variables	All
N	178
Sex (male %)	138 (77.53%)
Age (years)	10.4 ± 3.8
Bone age (years)	8.5 ± 4.1
Height (cm)	126.47 ± 19.62
Height SDS	−2.71 (−3.18--2.31)
Mother’s height (cm)	155.38 ± 5.54
Father’s height (cm)	167.59 ± 5.41
Body weight (kg)	27.29 ± 10.76
BMI (kg/m^2^)	16.38 ± 2.57
IGF-1 (ng/mL)	175.50 (90.05–273.75)
IGF-1 SDS	− 0.99 (− 1.60--0.09)
Hb (g/L)	131.81 ± 9.36
Cr (umol/L)	38.75 ± 9.75
BUN (umol/L)	4.65 ± 1.15
UA (umol/L)	268.55 ± 75.63
TG (mmol/L)	0.65 ± 0.24
TC (mmol/L)	3.79 ± 0.66
HDL (mmol/L)	1.35 ± 0.27
LDL (mmol/L)	2.07 ± 0.51
ALT(U/L)	15.68 ± 6.87
FPG (mmol/L)	4.80 ± 0.58
Estradiol (pg/mL)	17.83 (11.80–26.79)
Testosterone (ng/mL)	0.25 (0.10–0.86)
Pubertal stage	
Prepubertal (%)	129 (72.47%)
Pubertal (%)	49 (27.53%)

**Abbreviations:***Height SDS* height standard deviation scores, *BMI* body mass index, *IGF-1 SDS* insulin like growth factor-1 standard deviation scores, *Hb* hemoglobin, *Cr* creatinine, *BUN* blood urea nitrogen, *UA* uric acid, *TG* triglyceride, *TC* total cholesterol, *HDL-C* high density lipoprotein-cholesterol, *LDL-C* low density lipoprotein cholesterol, *ALT* alanine aminotransferase, *FPG* fasting plasma glucose

Normal distribution of data was presented as mean ± standard deviation; nonnormal distribution of data was presented as median (interquartile range) and categorical data using number (percentage)

**Table 2:** Association between IGF-1 SDS and different variables (*n* = 178)

Variables	B	(95% CI)	*P* value
Age (years)	0.10	(0.06, 0.15)	< 0.001
Bone age (years)	0.11	(0.07, 0.15)	< 0.001
Height SDS	0.60	(0.32, 0.89)	< 0.001
Mother’s height (cm)	−0.01	(−0.04, 0.03)	0.739
Father’s height (cm)	0.01	(−0.03, 0.04)	0.735
Body weight (kg)	0.06	(0.04, 0.07)	< 0.001
BMI (kg/m^2^)	0.20	(0.13, 0.26)	< 0.001
Hb (g/L)	0.04	(0.03, 0.06)	< 0.001
Cr (umol/L)	0.03	(0.01, 0.05)	< 0.001
BUN (umol/L)	−0.03	(−0.20, 0.13)	0.697
UA (umol/L)	0.01	(0.00, 0.01)	0.017
TG (mmol/L)	0.29	(−0.51, 1.09)	0.480
TC (mmol/L)	−0.20	(−0.50, 0.09)	0.177
HDL (mmol/L)	−0.36	(−1.08, 0.35)	0.316
LDL (mmol/L)	−0.25	(−0.63, 0.13)	0.193
ALT(U/L)	0.02	(−0.01, 0.05)	0.145
FPG (mmol/L)	0.41	(0.08, 0.74)	0.014
Estradiol (pg/mL)	0.02	(0.01, 0.03)	< 0.001
Testosterone (ng/mL)	0.35	(0.17, 0.53)	< 0.001
Sex			
Male	reference		
Female	−0.33	(−0.76, 0.11)	0.142
Pubertal stage			
Prepubertal (%)	reference		
Pubertal (%)	0.96	(0.55, 1.36)	< 0.001

**Abbreviations:***Height SDS* height standard deviation scores, *BMI* body mass index, *Hb* hemoglobin, *Cr* creatinine, *BUN* blood urea nitrogen, *UA* uric acid, *TG* triglyceride, *TC* total cholesterol, *HDL-C* high density lipoprotein-cholesterol, *LDL-C* low density lipoprotein cholesterol, *ALT* alanine aminotransferase, *FPG* fasting plasma glucose

B unstandardized regression coefficient. *P* < 0.05 is considered to be statistically signifcant

**Table 3:** Threshold effect analysis for the relationship between Hb and IGF-1 SDS

Models	IGF-1 SDS	
	Adjusted B (95%CI)	*P* value
Model I		
One line slope (*n =* 178)	0.03 (0.01, 0.05)	0.001
Model II		
Hemoglobin’s inflection point	145	
< 145 (*n* = 162)	0.05 (0.02, 0.07)	< 0.001
> 145 (*n =* 16)	−0.15 (− 0.23, − 0.06)	0.001
LRT test	0.044	

Model I, linear analysis; Model II, nonlinear analysis. LRT test, logarithmic likelihood ratio test (*p*-value< 0.05 indicates that Model II is significantly different from Model I, which indicates a nonlinear relationship); adjustment variables: age, sex, BMI, TC, pubertal stage

*BMI* body mass index, *TC* total cholesterol

B unstandardized regression coefficient; *P* < 0.05 was considered to be statistically significant

## Results

### Clinical and biochemical characteristics

The baseline characteristics of the participants are displayed in Table 1. A total of 178 children, consisting of 138 (77.53%) males and 40 (22.47%) females, were recruited. The mean or median values (interquartile range) of the anthropometric measurements and biomarkers among participants aged 10.4 ± 3.8 years old were described. The mean bone age of the subjects was 8.5 ± 4.1 years, and the median and interquartile range of height SDS was − 2.71 (− 3.18--2.31). A total of 129 (72.47%) subjects were prepubescent, and only 49 (27.53%) were pubescent. The mean levels of IGF-1 SDS and Hb were − 0.99 (− 1.60 - -0.09) and 131.81 ± 9.36 g/L, respectively.

### Factors associated with IGF-1 SDS in the subjects

Table 2 shows the associations between IGF-1 SDS and all tested variables by univariate analysis. A significant positive relationship between IGF-1 SDS and Hb was observed (*P* < 0.001). Other variables, including age, bone age, height SDS, weight, BMI, Cr, estradiol, testosterone, stage of puberty (all *P <* 0.001), UA (*P* = 0.017) and FPG (*P* = 0.014), were all positively associated with IGF-1 SDS.

### Independent association between IGF-1 SDS and Hb

As shown in Table 3, multiple regression was performed after adjustment for potential confounding factors based on the results of the univariate analysis, including age, height, weight, Cr, UA, FPG, estradiol, testosterone, and stage of puberty, to further analyze the independent association between IGF-1 SDS and Hb. The positive relationship between Hb and IGF-1 SDS remained (B 0.03; 95% CI 0.01, 0.05; *P* = 0.001). Smooth curve fitting was performed after adjustment for potential confounding factors, and a nonlinear relationship was observed between IGF-1 SDS and Hb. A two-stage change and an inflection point were displayed in the resultant curve (Fig. 2a). A scatter plot of the distribution of child Hb and IGF-1 SDS was presented in Fig. 2b. Specifically, there was a positive association between IGF-1 SDS and Hb when the concentration of Hb was lower than the turning point. However, there was a negative association between IGF-1 SDS and Hb when the concentration of Hb was beyond the turning point. In addition, we further applied multivariate piecewise linear regression to evaluate the independent relationship between IGF-1 SDS and Hb in line with smooth curve fitting, and the inflection point was 145 g/L (Table 3). The analysis of threshold effects indicated that IGF-1 SDS increased with the elevation of Hb when the Hb concentration was lower than 145 g/L, for which the mean concentration of Hb was 130.02 ± 7.58 g/L (B 0.05; 95% CI 0.02, 0.07; *P* < 0.001), which approximates the normal value recommended by the literature [23]; however, IGF-1 SDS decreased with Hb elevation when the Hb concentration was greater than 145 g/L, for which the mean concentration of Hb was 149.94 ± 5.58 g/L (B -0.15; 95% CI -0.23, − 0.06; *P* = 0.001). In addition, we used the original IGF-1 data to adjust for age and sex and performed an internal analysis between Hb and IGF-1. We found that the results were consistent with the relationship between Hb and IGF-1 SDS (data not shown).

## Discussion

A nonlinear relationship was observed between IGF-1 SDS and Hb in children with ISS. More specifically, our study found that IGF-1 SDS increased as Hb increased when the Hb concentration was lower than 145 g/L. However, when the Hb concentration further increased, there was a negative association between IGF-1 SDS and Hb.

There is a large number of children and adolescents with short stature in China. An epidemiological study indicated that the incidence of primary and middle school students with short stature in China is approximately 3.16% [24], but there is still a lack of systematic studies. Furthermore, the pathogenesis of short stature in children and adolescents is understudied. We established a clinical cohort of short stature patients (GDDSD) with the main purpose of exploring the association of clinical indicators in these patients and to provide new insights and evidence for the etiology and subsequent diagnosis of short stature.

This study found a positive relationship between IGF-1 SDS and Hb when Hb concentrations were lower than 145 g/L, which was consistent with previous findings in other populations [17–19, 25, 26]. Succurro E et al. [17] conducted a study in adult nondiabetic subjects and showed that IGF-1 levels were positively associated with Hb concentration. However, their study was only based on Caucasian subjects, and the results in other ethnic groups need further exploration. Francesca De Vita et al. [25] demonstrated that the levels of IGF-1 were independent and positively related to Hb concentration in aged populations. A recent study [19] reported a positive association of IGF-1 concentration with Hb using a linear regression model in prepubescent children with PA. Vihervuori E et al. conducted a Pearson’s correlation analysis in 32 children and described a positive correlation between Hb and serum IGF-1 levels [26]. Moreover, an observational study conducted in prepubescent children showed that IGF-1 was significantly lower in an anemic group than in a control group [27]. Additionally, an intervention study demonstrated that short-term treatment with iron in children with anemia significantly increased circulating IGF-1 concentrations [28].

Interestingly, we further analyzed the relationship between Hb and IGF-1 through smooth curve fitting and observed a nonlinear relationship between Hb and IGF-1 SDS. The levels of IGF-1 increased with increasing Hb, and when the concentration of Hb exceeded a certain range, with the increase in Hb, IGF-1 decreased instead. Previous studies have shown that high Hb may impact the growth and development of children through mechanisms including inflammation, which may impair growth and development [16]. Our findings suggested that a too high of Hb is associated with reduced IGF-1 levels.

The potential mechanisms of this phenomenon can be explained as follows: low oxygen conditions at low Hb concentrations inhibit IGF-1 action through an increase in phosphorylated IGF binding protein-1 (IGFBP-1), which inhibits IGF-I action [29]. Tran Phu V et al. demonstrated in a dietary-induced rat model that gestational anemia attenuates postnatal hippocampal IGF signaling, and hippocampal IGF activation was markedly suppressed [30]. High Hb concentrations may function through induced oxidative stress, increased blood viscosity, and impaired systemic response to inflammation and infection [13–15] to further impact the linear growth of children. However, the exact mechanism by which high Hb is associated with low IGF-1 is unclear. Basic experiments are needed to further explore the underlying mechanism of the relationship between high Hb and low IGF-1.

Because IGF-1 standardization varies, to increase the authenticity and reliability of our study results, we used the original IGF-1 data to perform an internal analysis, and the relationship between Hb and IGF-1 remained stable and reliable. However, this study has some limitations. First, due to the cross-sectional analysis, no definitive causative relationship can be inferred in the study. Second, the present findings are only based on children with ISS, and different results might be observed in other groups, such as children with GH deficiency. Third, these children are short in stature for unknown reasons and have no other diseases, so the amount of high Hb is limited, especially in prepubertal children. Finally, although we excluded children who received medication that interferes with GH secretion or its effects, the history of previous medications was reported by the parents of the children themselves, which may not be accurate.

## Conclusion

We described a nonlinear relationship between Hb and IGF-1 in Chinese children and adolescents with ISS. IGF-1 levels increased with the elevation of Hb concentration; when the increment of Hb exceeded a certain range, IGF-1 decreased instead.

## Data Availability

The data used to support the findings of this study are available from the corresponding author upon request.

## Abbreviations

IGF-1: Insulin-like growth factor-1
ISS: Idiopathic short stature
Height SDS: Height standard deviation scores
BMI: Body mass index
Hb: Hemoglobin
Cr: Creatinine
BUN: Blood urea nitrogen
UA: Uric acid
TG: Triglyceride
TC: Total cholesterol
HDL-C: High density lipoprotein – cholesterol
LDL-C: Low density lipoprotein cholesterol
ALT: Alanine aminotransferase
FPG: Fasting plasma glucose
